# Disruptive influence

**DOI:** 10.7554/eLife.01779

**Published:** 2013-12-03

**Authors:** Giulio Cossu, Artal Moreno-Fortuny, Urmas Roostalu

**Affiliations:** 1 **Giulio Cossu** is an *eLife* reviewing editor and is at the Institute of Inflammation and Repair, University of Manchester, Manchester, United Kingdomgiulio.cossu@manchester.ac.uk; 2 **Artal Moreno-Fortuny** is at the Institute of Inflammation and Repair, University of Manchester, Manchester, United Kingdomartalmf@gmail.com; 3 **Urmas Roostalu** is at the Institute of Inflammation and Repair, University of Manchester, Manchester, United Kingdomurmas.roostalu@manchester.ac.uk

**Keywords:** Rb, CDK, phosphorylation, sarcomeric organization, retinoblastoma protein, Human

## Abstract

Cachexia, a condition that kills about one-fifth of cancer patients, may be linked to Rb—a protein that is already linked to various cancers—moving from the cell nucleus to the cytoplasm.

**Related research article** Araki K, Kawauchi K, Hirata H, Yamamoto M, Taya Y. 2013. Cytoplasmic translocation of the retinoblastoma protein disrupts sarcomeric organization. *eLife* **2**:e01228. doi: 10.7554/eLife.01228

**Image** Confocal images showing phosphorylated Rb (red) and cell nuclei (blue); most of the Rb is not localized in the nuclei of myotubes that have been treated (right). Scale bar is 20 microns

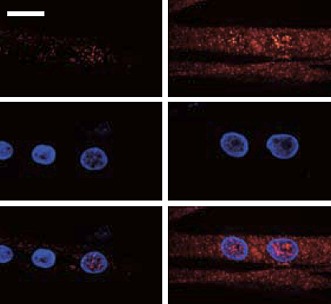

With more than 11,000 papers published on it, one would assume that we know all there is to know about retinoblastoma protein (Rb). This protein has been the subject of intense study ever since it was realized that the inactivation of Rb is a fundamental event in the development of cancer. However, it has been difficult to determine what Rb does in normal cells because it appears to be involved in a wide range of processes, including transcriptional regulation, chromatin remodelling, the cell cycle and apoptosis. Now, in *eLife*, Keigo Araki, Yoichi Taya and colleagues at the National University of Singapore report an intriguing new aspect of Rb. They show that, under certain conditions, Rb can move from the cell nucleus—where it is usually found—to the cytoplasm, where it disrupts the basic units of muscle (Araki et al., 2013).

This finding is important because it could help explain why late-stage cancer patients often encounter progressive muscle weakness and atrophy that cannot be reversed by increased calorie intake (reviewed in Fearon et al., 2012). It has been estimated that this condition—which is called cachexia, and which also involves changes to the neuroendocrine system, the immune system and adipose tissue–is the direct cause of death for approximately 20% cancer patients, so obtaining a better understanding of the molecular mechanisms responsible for it is of paramount importance. According to the current understanding of cachexia, various inflammatory cytokines lead to the up-regulation of enzymes called E3 ubiquitin ligases, which then mediate the breakdown of the proteins that make up sarcomeres, which are the basic building blocks of muscle. At the same time, the expression of the genes that code for skeletal muscle are down-regulated.

The new results demonstrate that Rb can destabilize the sarcomeric proteins in the muscle cells of cancer patients: by interacting with mDia1 (a protein that nucleates the formation of actin filaments in the cytoplasm), Rb may influence both the assembly of actin filaments and the generation of force by these filaments (Figure 1). It remains to be seen whether the interaction between mDia1 and Rb is the consequence of the activity of E3 ligases or of altered cellular energy balance, or if it precedes these stages in the weakening of skeletal muscle. Indeed, it has been suggested that the filaments are more susceptible to degradation after they have been dislodged from the sarcomere (Solomon and Goldberg, 1996). The equivalent of mDia1 in *C. elegans* is known to be important in the attachment of actin filaments to a structure called the Z-line, which marks the edge of the sarcomere (Figure 1; Mi-Mi et al., 2012). It is conceivable, therefore, that the co-localization of mDia1 and Rb near the Z-line in human cells may lead to the degradation of actin filaments and enhance the breakdown of proteins within the cells.

**Figure 1.**
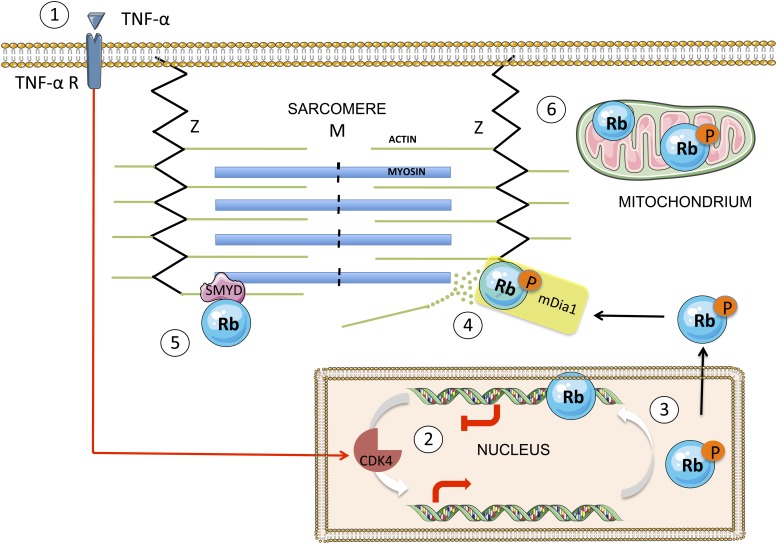
A simplified scheme showing the translocation of Rb from the nucleus to the cytoplasm, and the effect it has on sarcomeres. TNF-α (tumour necrosis factor-alpha) is a cytokine that may, in certain cases, kill cancer cells. When TNF-α binds to its receptor on the surface of a normal cell (1), it triggers a signal transduction cascade that leads to the phosphorylation of Rb in the nucleus (2) by the enzyme CDK4. The phosphorylated Rb (3) separates from the DNA, allowing transcription of E2F target genes, and moves to the cytoplasm, where it binds to mDia1 at the Z line (4). This leads to the destabilization of the actin filaments in the sarcomere and to impaired muscle function. The enzyme SMYD methylates the Rb, which contributes to sarcomere stability (5). There is also evidence that Rb accumulates in the mitochondria, where it seems to play a role in apoptosis (6). The figure was produced using Servier Medical Art (http://www.servier.com).

A considerable amount of evidence about the diverse range of roles performed by Rb in the cytoplasm has emerged in recent years. For example, it has been demonstrated that Rb can be localized in mitochondria and also shown that it is involved in inducing apoptosis (Hilgendorf et al., 2013). Moreover, in various cells Rb is methylated by an enzyme called SMYD2, which enhances the phosphorylation of Rb and the recruitment of additional co-factors (Saddic et al., 2010; Cho et al., 2012). And in separate studies of skeletal muscle cells, SMYD1 and SMYD2 have been found to be vital for forming complexes with myosin, myosin chaperones and titin. Loss of SMYD function is also known to lead to the disruption of sarcomere (Just et al., 2011; Donlin et al., 2012; Li et al., 2013).

These data place Rb in a protein interaction network, which now includes both myosin and actin filaments of skeletal muscle, along with their regulatory factors. We can expect exciting times in unravelling how this stress responsive pathway is fine-tuned to sense the balance between healthy cells, sarcomere breakdown and apoptosis. It will be intriguing to study how these cytoplasmic functions of Rb relate to its role as a transcriptional regulator in the nucleus and how Rb is distributed between different cellular compartments.

With these newly identified functions, Rb has managed to surprise us once again. The loss of Rb has long been associated with cancer. Now it appears that native Rb can also have detrimental effects for patients away from cancer tissue. And while the presence of Rb in the cytoplasm of cells in skeletal muscles can have damaging consequences, we still do not know if there is an underlying physiological role for Rb in the healthy turnover sarcomeres. Ultimately, of course, we hope it will be possible to minimize cancer-related muscle wasting by modifying the behaviour of Rb in skeletal muscle cells.

## References

[bib1] Araki K Kawauchi K Hirata H Yamamoto M Taya Y 2013 Cytoplasmic translocation of the retinoblastoma protein disrupts sarcomeric organization. eLife 2:e01228. 10.7554/eLife.01228 PMC3843810 24302570

[bib2] Cho H Hayami S Toyokawa G Maejima K Yamane Y Suzuki T Dohmae N Kogure M Kang D Neal DE Ponder BA Yamaue H Nakamura Y Hamamoto R 2012 RB1 methylation by SMYD2 enhances cell cycle progression through an increase of RB1 phosphorylation. Neoplasia 14:476–486. 10.1593/neo.12656 22787429 PMC3394190

[bib3] Donlin LT Andresen C Just S Rudensky E Pappas CT Kruger M Jacobs EY Unger A Zieseniss A Dobenecker MW Voelkel T Chait BT Gregorio CC Rottbauer W Tarakhovsky A Linke WA 2012 Smyd2 controls cytoplasmic lysine methylation of Hsp90 and myofilament organization. Genes & Development 26:114–119. 10.1101/gad.177758.111 22241783 PMC3273835

[bib4] Fearon KC Glass DJ Guttridge DC 2012 Cancer cachexia: mediators, signaling, and metabolic pathways. Cell Metab 16:153–166. 10.1016/j.cmet.2012.06.011 22795476

[bib5] Hilgendorf KI Leshchiner ES Nedelcu S Maynard MA Calo E Ianari A Walensky LD Lees JA 2013 The retinoblastoma protein induces apoptosis directly at the mitochondria. Genes Dev 27:1003–1015. 10.1101/gad.211326.112 23618872 PMC3656319

[bib6] Just S Meder B Berger IM Etard C Trano N Patzel E Hassel D Marquart S Dahme T Vogel B Fishman MC Katus HA Strähle U Rottbauer W 2011 The myosin-interacting protein SMYD1 is essential for sarcomere organization. Journal of Cell Science 124:3127–3136. 10.1242/jcs.084772 21852424

[bib7] Li H Zhong Y Wang Z Gao J Xu J Chu W Zhang J Fang S Du SJ 2013 Smyd1b is required for skeletal and cardiac muscle function in zebrafish. Molecular Biology of the Cell 24:3511–3521. 10.1091/mbc.E13-06-0352 24068325 PMC3826989

[bib8] Mi-Mi L Votra S Kemphues K Bretscher A Pruyne D 2012 Z-line formins promote contractile lattice growth and maintenance in striated muscles of *C. elegans*. The Journal of Cell Biology 198:87–102. 10.1083/jcb.201202053 22753896 PMC3392944

[bib9] Saddic LA West LE Aslanian A Yates JR Rubin SM Gozani O Sage J 2010 Methylation of the retinoblastoma tumor suppressor by SMYD2. The Journal of Biological Chemistry 285:37733–37740. 10.1074/jbc.M110.137612 20870719 PMC2988378

[bib10] Solomon V Goldberg AL 1996 Importance of the ATP-ubiquitin-proteasome pathway in the degradation of soluble and myofibrillar proteins in rabbit muscle extracts. The Journal of Biological Chemistry 271:26690–26697. 10.1074/jbc.271.43.26690 8900146

